# Triplet State Suppression for Energy Loss Reduction in 20% Nonhalogenated Solvent Processed Binary Organic Solar Cells

**DOI:** 10.1002/adma.202500861

**Published:** 2025-03-16

**Authors:** Ruijie Ma, Bosen Zou, Yulong Hai, Yongmin Luo, Zhenghui Luo, Jiaying Wu, He Yan, Gang Li

**Affiliations:** ^1^ Department of Electrical and Electronic Engineering Research Institute for Smart Energy (RISE) Photonic Research Institute (PRI) The Hong Kong Polytechnic University Hong Kong 999077 China; ^2^ Department of Chemistry Department of Chemistry and Hong Kong Branch of Chinese National Engineering Research Center for Tissue Restoration and Reconstruction The Hong Kong University of Science and Technology Clear Water Bay Hong Kong 999077 China; ^3^ The Hong Kong University of Science and Technology (Guangzhou) Function Hub Advanced Materials Thrust Nansha Guangzhou 511400 China; ^4^ Guangdong Provincial Key Laboratory of New Energy Materials Service Safety Shenzhen Key Laboratory of New Information Display and Storage Materials College of Materials Science and Engineering Shenzhen University Shenzhen 518060 China

**Keywords:** energy loss, nonhalogenated solvent, organic solar cell, power conversion efficiency, triplet state suppression

## Abstract

Boosting power conversion efficiency (PCE) of organic solar cells (OSCs) has been restricted by its undesirably high energy loss, especially for those nonhalogenated solvent‐processed ones. Here,a dichloro‐methoxylated terminal group in an asymmetric small molecular acceptor design, which realizes a significantly reduced non‐radiative energy loss (0.179 eV) compared to its symmetric counterpart (0.202 eV), is reported. Consequently, the device efficiency is improved by up to 20% for PM6:BTP‐eC9‐4ClO, without sacrificing the photon harvest or charge transport ability of the control system PM6:BTP‐eC9. Further characterizations reveal the asymmetric acceptor BTP‐eC9‐4ClO's blend film demonstrates a suppressed triplet state formation, enabled by an enhanced electron delocalization. In addition, the asymmetric BTP‐eC9‐4ClO is found to be thermally stabler than BTP‐eC9, and thus providing an improved device stability, whose T80 value reaches > 7800 h under 80 °C anneal in N_2_ via linear extrapolation. This work represents state‐of‐the‐art device performance for nonhalogenated solvent‐processed binary OSCs with certified results (19.45%).

## Graphical abstract

Asymmetric small molecule acceptor with a methoxylated end group is produced here, whose oxygen vibration is found effective in suppressing triplet state formation, thereby minimizing non‐radiative energy loss for 20% efficiency in nonhalogenated solvent cast binary organic solar cells.

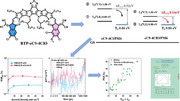

## 1 Introduction

Organic solar cells (OSCs) with color tunability, modulable absorption spectra, low‐cost, and lightweight properties are of promising marketing value.^[^ ^1^, ^2^, ^3^, ^4^, ^5^, ^6^ ^]^ However, limited by organic semiconductor's insufficient crystallinity, inevitable vibronic coupling, and triplet state formation, the power conversion efficiency (PCE) of OSCs has been lagged behind compared to traditional inorganic solar cells, due to high energy loss,^[^ ^7^, ^8^, ^9^, ^10^, ^11^, ^12^ ^],^ especially for nonhalogenated solvent (representative solvent: *ortho*‐xylene [*o*‐XY]) processed OSCs.^[^ ^13^, ^14^, ^15^, ^16^, ^17^, ^18^, ^19^, ^20^ ^]^ Thus, realizing a more reduced energy loss through suppressing undesired exciton transition processes is the key issue for further boosting PCE.

In the past few years, constructing asymmetric small molecular acceptor (SMA) through terminal group modification has been developed as a widely acknowledged strategy to achieve reduced energy loss and kept charge behavior.^[^ ^21^, ^22^, ^23^, ^24^, ^25^, ^26^, ^27^ ^]^ For example, Chen's group has achieved a series of performance progress by designing asymmetric SMAs, that are effective in both binary and ternary OSCs.^[^ ^28^, ^29^, ^30^, ^31^ ^]^ Meanwhile, our team has also reported some cases on this topic.^[^ ^32^, ^33^, ^34^ ^]^ However, the sacrificed absorption range has not been overcome in these works, thus decreasing short‐circuit current density (*J*_SC_). In addition, the underlying mechanism of energy loss reduction has not been clearly revealed in the view of molecular structure. Last but not least, the performance of binary OSCs based on asymmetric SMAs is still < 20%, for both halogenated solvent and nonhalogenated solvent processed devices.

In this work, we proposed a new asymmetric SMA derived from BTP‐eC9 (high PCE in *o*‐XY processed devices) through a mature methoxylated terminal group construction, named BTP‐eC9‐4ClO.^[^ ^14^, ^33^ ^]^ It has a significantly larger dipole moment than BTP‐eC9 as expected, yet a similar bandgap is evidenced by calculation and experiment. Rationally, BTP‐eC9‐4ClO exhibits a more ordered crystalline framework and similar aggregation tendency. For blend films, the crystalline structure of PM6:BTP‐eC9‐4ClO system's *π*–*π* stacking is more ordered, while it demonstrates a reduced acceptor‐rich domain size, that is beneficial to charge generation, facilitated by its slightly longer exciton diffusion length. Moreover, a suppressed triplet formation in the PM6:BTP‐eC9‐4ClO active layer is observed, which is explained by theoretical analysis: enhanced electron delocalization results in dynamic equilibrium between charge transfer state and triplet state. As a consequence, a significantly reduced non‐radiative energy loss is achieved, resulting in a 20% efficiency binary OSC cast by nonhalogenated solvent *o*‐XY, with a certified PCE of 19.45%. Parallelly, BTP‐eC9‐4ClO shows better thermal stability, yielding an over 7800 h (estimated) *T*_80_ value under 80 °C stress in N_2_ atmosphere. Overall, our report presents a highly efficient and stable OSC enabled by asymmetric SMA design, with a detailed and in‐depth mechanism study on energy loss suppression.

## 2 Results and Discussion

**Figure 1:**
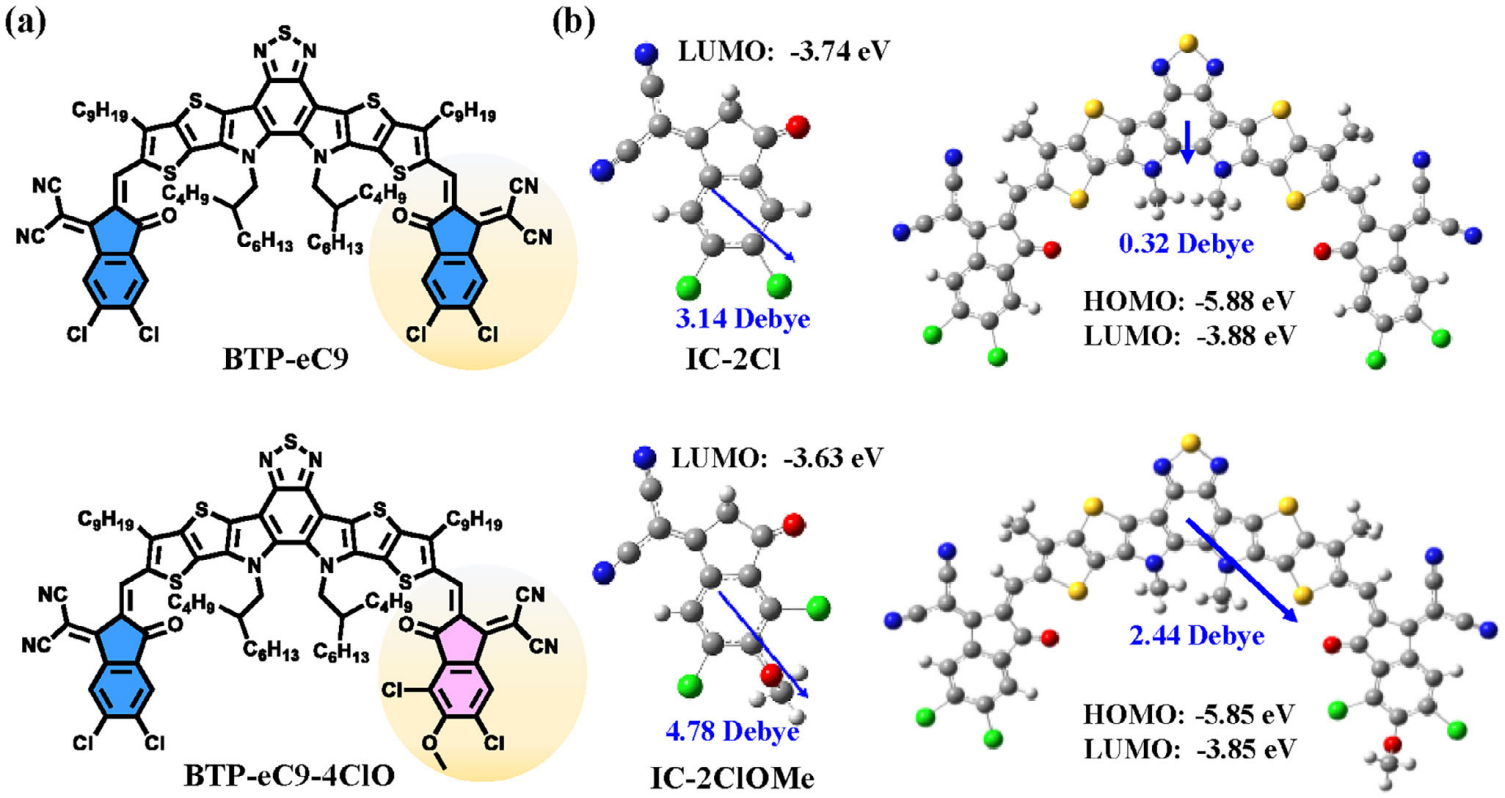
a) Chemical structures of studied materials. b) DFT calculation results on 2 molecules.

The chemical structures of BTP‐eC9 and BTP‐eC9‐4ClO are presented in **Figure** **1**a, with highlighted terminal groups for a clear comparison. The synthetic route of BTP‐eC9‐4ClO is given in Supporting Information. The nuclear magnetic resonance (NMR) spectra and mass spectra for the new terminal group IC‐2ClOMe, and the new acceptor are presented from Figures S2–S7 (Supporting Information). Meanwhile, the thermogravimetric analysis (TGA) is applied on BTP‐eC9‐4ClO, whose decomposition temperature (*T*_d_) is found 311 °C.

Then the basic density function theory (DFT) calculation on two terminal groups and SMAs at the non‐empirically tuned B3LYP‐D3(BJ)/TZVP level of theory is utilized.^[^ ^35^, ^36^, ^37^, ^38^ ^]^ As depicted by Figure 1b, IC‐2ClOMe possesses a dipole moment of 4.78 Debye, larger than that of IC‐2Cl (3.74 Debye). The symmetry breaking at the terminal group also leads to an enlarged dipole moment from 0.32 to 2.44 Debye for BTP‐eC9‐4ClO, which has been reported as potentially beneficial to improving exciton dissociation.^[^ ^39^ ^]^ On the other hand, the assessed lowest unoccupied molecular orbital (LUMO) levels of two terminal groups are proposed at −3.74  and −3.63 eV, indicating the weaker electron‐withdrawing ability of IC‐2ClOMe than IC‐2Cl. The LUMO and highest occupied molecular orbital (HOMO) level distributions are then (−3.88 eV, −5.88 eV) and (−3.85 eV, −5.85 eV) for BTP‐eC9 and BTP‐eC9‐4ClO, respectively. Thereby, the energy level distribution is not significantly altered by our asymmetric modification. More to see in Figures S13–S16 (Supporting Information).

**Figure 2:**
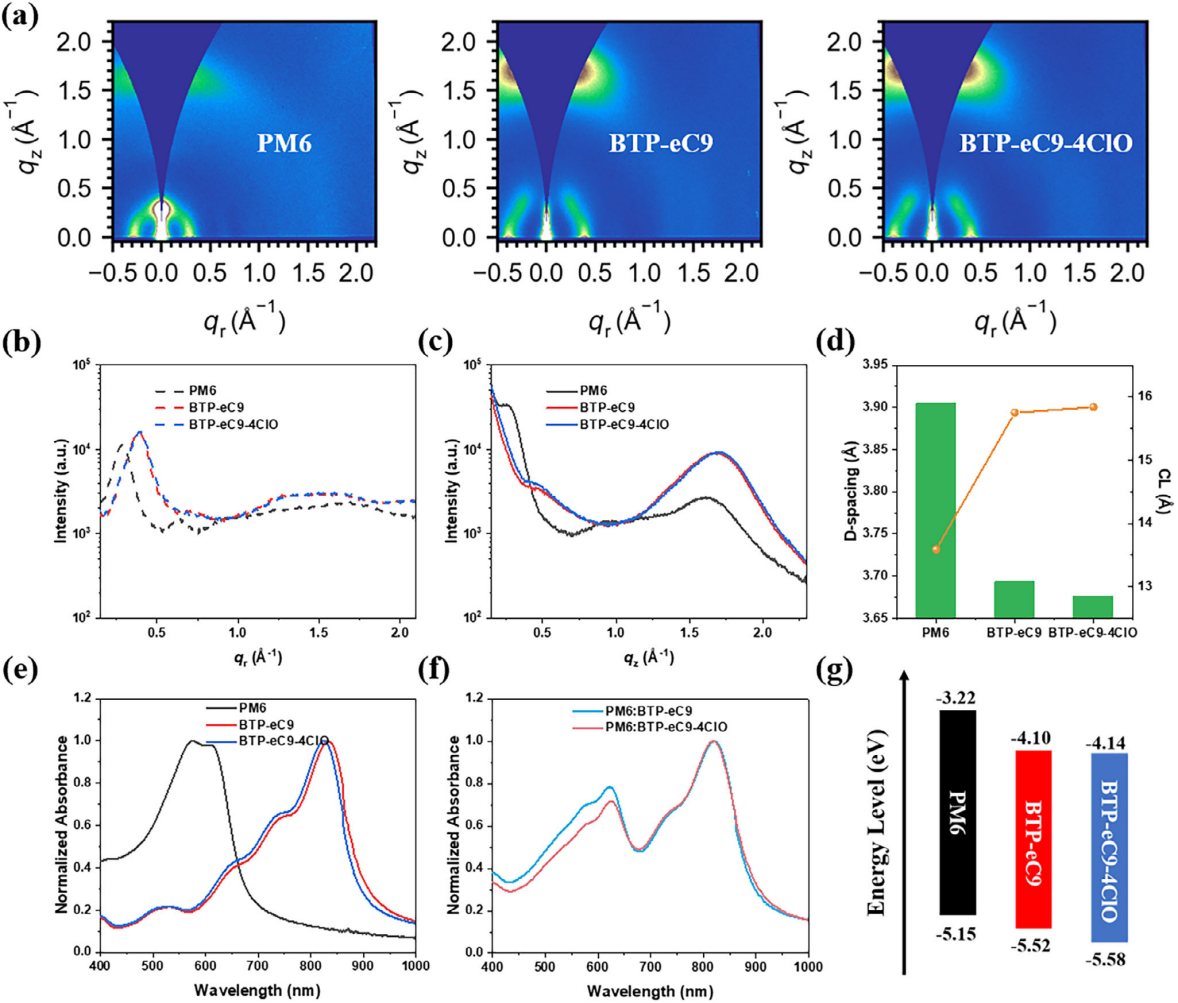
a) 2D‐GIWAXS patterns of PM6, BTP‐eC9, and BTP‐eC9‐4ClO neat films. b) IP and c) OOP line‐cuts. d) Crystalline parameters of *π*–*π* stacking peaks. UV–vis spectra of e) pure films and f) blend films. g) Energy level distribution of neat films determined by UPS results.

Next, the crystalline characteristics of studied materials in film state are investigated by grazing incidence wide‐angle X‐ray scattering (GIWAXS) experiments.^[^ ^40^, ^41^, ^42^, ^43^, ^44^ ^]^ The 2D patterns are put in **Figure** **2**a, with their in‐plane (IP) and out‐of‐plane (OOP) line cuts plotted in Figure 2b,c. The fitted crystalline parameters including peak position, d‐spacing values, and coherence length (CL) are displayed in Tables S1 and S2 (Supporting Information), and for OOP *π*–*π* stacking signals, the d‐spacing and CL value variation is visualized by Figure 2d, as well. The characteristic peaks for PM6 are at 0.29 and 1.61 Å^−1^, for (100) and (010) peaks, while those for BTP‐eC9 and BTP‐eC9‐4ClO are (0.39 Å^−1^, 1.70 Å^−1^) and (0.39 Å^−1^, 1.71 Å^−1^). These differences are helpful in analyzing the blend film crystalline features. In addition, the crystallization characteristics of the two acceptors are very similar. Then, the UV–vis absorption spectra are measured for pure and blend films, as displayed in Figure 2e,f. Accordingly, two acceptors demonstrate similar absorption range and aggregation motifs, though BTP‐eC9‐4ClO has a slightly blue‐shift edge. Besides, the optical bandgaps of the neat films can be assessed as 1.93, 1.42, and 1.44 eV for PM6, BTP‐eC9, and BTP‐eC9‐4ClO. Thus, combing with the ultraviolet photoelectron spectroscopy (UPS) characterization (Figure S9, Supporting Information) determined HOMO levels, the energy landscape of three materials is drawn in Figure 2g. The LUMO and HOMO levels shift from (−4.10 eV, −5.52 eV) of BTP‐eC9 to (−4.14 eV, −5.58 eV) of BTP‐eC9‐4ClO. Both two materials are supposed to match well with PM6, whose energy level is located at c.a. (−3.22 eV, −5.15 eV).

**Figure 3:**
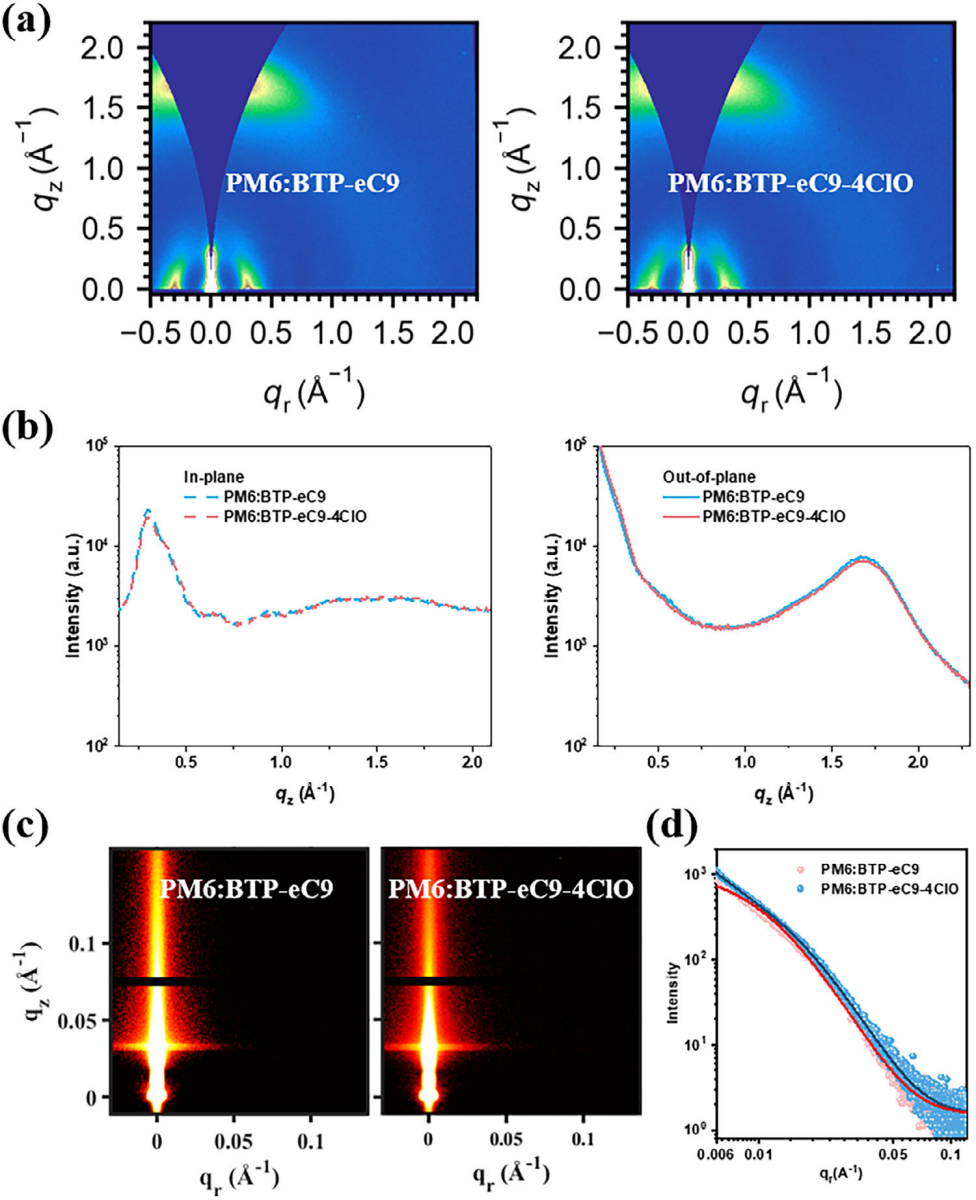
a) 2D‐GIWAXS patterns of PM6:BTP‐eC9 and PM6:BTP‐eC9‐4ClO blend films. b) Corresponding IP and OOP line‐cuts. c) 2D‐GISAXS patterns. d) Extracted IP directional intensity profiles and fitting lines.

Then the blend morphology (crystallization feature and phase segregation) is studied through a series of experiments. The 2D GIWAXS patterns and corresponding line cuts are depicted in **Figure** **3**a,b. Likewise, their analyzed crystalline parameters are summarized in Tables S3 and S4 (Supporting Information). In blend films, the IP directional lamellar packing is dominated by PM6, as the peak locations reside where PM6's signal contributes. On the other hand, the *π*–*π* stacking peaks alongside the OOP direction are of the acceptor's motif, which is also evidenced by diffraction peak locations. Both two active layers are of the typical face‐on orientation, suggesting favorable charge transport properties. The packing ordering (CL values) of (100) peak suggests PM6:BTP‐eC9‐4ClO has a more desirable long‐distance packing, and its (010) peak signal also marginally outperforms those of PM6:BTP‐eC9, implying boosted charge transport, and thereby suppressed free charge recombination.^[^ ^45^, ^46^, ^47^ ^]^

Subsequently, the grazing incidence small angle X‐ray scattering (GISAXS) test is applied, too.^[^ ^48^, ^49^, ^50^ ^]^ Figure 3c,d includes corresponding 2D patterns and IP directional line‐cuts, as well as fitting lines. Following the Debye–Anderson–Brumberger model, phase separation length scale parameters are extracted and presented in **Table** **1**, in which *ξ* is the domain size of the PM6‐rich region, D is aggregation dimensionality (set to be 3), *η* is correlation length, and 2*R*_g_ represents acceptor‐rich phase's length scale. Both systems demonstrate suitable donor and acceptor domain sizes that are beneficial to concurrently boosting charge generation and transport.

**Table 1:** Phase separation parameters.

PM6:acceptor	*ξ* [nm]	*η* [nm]	D	2*R*_g_ [nm]
BTP‐eC9	15.8	6.4	3	31.2
BTP‐eC9‐4ClO	18.6	4.3	3	21.1

In addition, the atomic force microscopy (AFM) measurement is carried out to gain a more direct impression of nano/micro‐scale morphology. As demonstrated by Figure S11 (Supporting Information), the fibril network occurs in both systems, implying similar general morphology is shared. On the contrary, the fiber size of PM6:BTP‐eC9‐4ClO is slightly larger, which could facilitate the charge transport.

**Figure 4:**
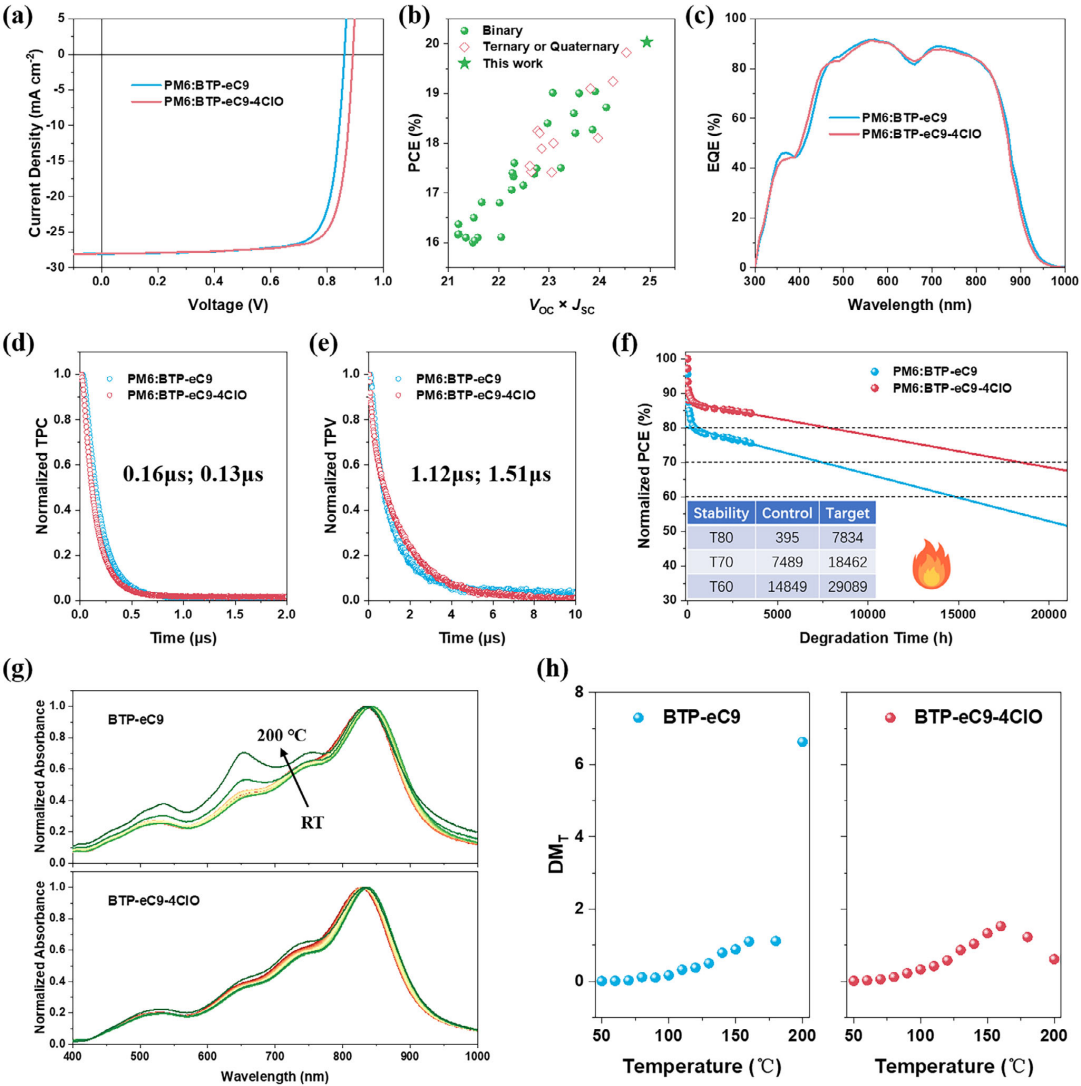
a) *J*–*V* characteristics b) Performance summary. c) EQE spectra. d) TPC and e) TPV results after normalization. f) Thermal stability. g) Temperature cascaded absorption profiles of two acceptors in film state. h) Calculated deviation metrics.

Then the photovoltaic performance is evaluated by fabricating a series of cells based on traditional architecture: ITO/Me‐4PACz/PEDOT: PSS/active layer/PFN‐Br/Ag.^[^ ^51^ ^]^ The current density versus voltage (*J–V*) characteristics of the two systems are plotted in **Figure** **4**a, whose photovoltaic parameters are presented in **Table** **2**. The processing solvent is selected as *o*‐XY, a high boiling point nonhalogenated solvent, to demonstrate more fabrication potential of our material system. Meanwhile, the additive treatment is enabled by a recently reported solid one BDCB (2‐monobromo‐1,3‐dichloro‐bezene).^[^ ^52^ ^]^ Compared to BTP‐eC9, the binary cell based on BTP‐eC9‐4ClO shows significantly improved *V*_OC_, and slightly increased fill factor (*FF*), resulting in boosted PCE to 20.03%. Besides, the averaged values of photovoltaic parameters are presented in Table 2 and Figure S11 (Supporting Information). This result is also certified by an independent institution (Figure S12, Supporting Information), where the *FF* suffers some reduction due to device degradation. Notably, the high efficiency yielded by PM6:BTP‐eC9‐4ClO stands for state‐of‐the‐art nonhalogenated solvent‐processed OSCs.^[^ ^53^, ^54^, ^55^ ^]^ This significance is also visualized in Figure 4b, for which the details of other literatures are provided in Table S5 (Supporting Information). In addition, the light absorbing range and *J–V* measurement accuracy are checked by their external quantum efficiency (EQE) spectra (Figure 4c). There exists no significant absorption range shift between the two blends, consistent with former analyses, and the *J*_SC_ mismatch is within 5%, a widely acknowledged value.

**Table 2:** Device performances.

PM6:acceptor	*V*_OC_ [V]	*J*_SC_ [mA cm^−2^]	*FF* [%]	PCE [%]
BTP‐eC9	0.861 (0.858 ± 0.003)	28.08/26.98	79.1 (78.8 ± 0.4)	19.12 (18.88 ± 0.21)
BTP‐eC9‐4ClO	0.891 (0.886 ± 0.004)	27.99/26.80	80.3 (79.8 ± 0.4)	20.03 (19.67 ± 0.32)
*Certified*	0.894	27.42	79.3	19.45

The brackets contain averages and standard errors of PCEs based on 20 devices. The integrated *J*_SC_s from EQE spectra are put behind the slashes.

The recombination dynamics are then analyzed by transient photocurrent (TPC) and transient photovoltage (TPV) to have a first glance the *FF* change (Figure 4d,e). The results initially suggest faster charge extraction and lower recombination in BTP‐eC9‐4ClO‐based devices. A more in‐depth discussion will be given later. The stability property of the two systems is compared by placing fresh inverted devices on 80 °C hotplate in N_2_. The results after tracking 1000 h and extrapolation analysis are depicted in Figure 4f. The calculated *T*_80_, *T*_70_, and *T*_60_ values are also put there. It suggests that the BTP‐eC9‐4ClO‐based device represents much stabler OSCs than those based on BTP‐eC9, whose *T*_80_ value is estimated over 7800 h and *T*_60_ value is predicted to approach 30 000 h, which appeals the advanced progress of OSCs.^[^ ^56^, ^57^, ^58^ ^]^ The stability promotion is simultaneously realized by reduced burn‐in loss and better long‐term performance maintenance. To further understand the thermal stability improvement brought by the new SMA, the deviation metrics are studied as shown in Figure 4g,h.^[^ ^59^, ^60^, ^61^, ^62^ ^]^ Tracing from room temperature to 200 °C, BTP‐eC9 and BTP‐eC9‐4ClO exhibit close glass transition temperatures (*T*_g_), but BTP‐eC9 has a larger variation slope at high‐temperature regions. Considering it as an accelerated degradation experiment, it can explain the elongated lifetime of PM6:BTP‐eC9‐4ClO‐based devices under long‐term thermal degradation. In addition, the reduced burn‐in loss of BTP‐eC9‐4ClO‐based binary device can be taken as a progress yielded by less separated initial morphology, as demonstrated by GISAXS analysis. Its smaller pure phase length scale is beneficial to minimize undesirable domain purification, which has been proposed to be one of the main factors of burn‐in degradation.^[^ ^63^ ^]^

**Figure 5:**
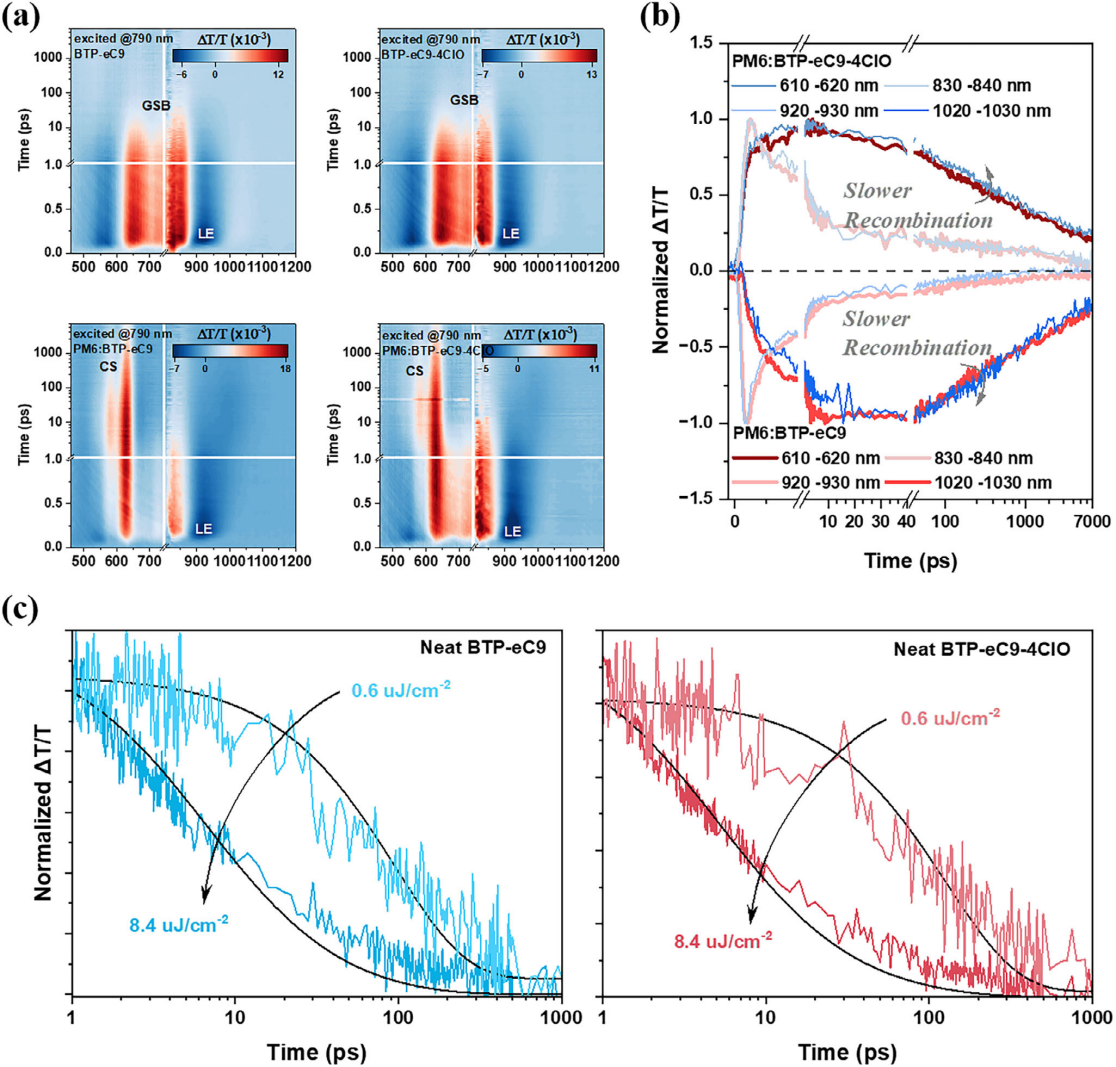
a) 2D contour maps of TAS measurements on neat and blend films. b) The extracted singleton and polaron kinetics. c) *L*_D_ calculation of acceptors via EEA model.

Next, more in‐depth charge behavior investigation is realized by the femto‐second transient absorption spectroscopy (fs‐TAS).^[^ ^64^, ^65^, ^66^ ^]^ The 2D contour maps of measurement results on neat acceptor films and blend films are presented in **Figure** **5**a, while the time scale spectral lines are placed in Figure S13 (Supporting Information). After we excited the pure acceptor with 780 nm light, we can clearly observe the exciton generation and recombination from Figure 5a (first two plots) with the rising of ground state bleaching (GSB, ≈620–880 nm) and photo‐induced absorption (PIA, ≈ 450–620 nm and ≈ 880–1000 nm). When we excited the acceptor of D:A blend, the excitons will generated first since we observed the faster rising of localized exciton at a range of 880–950 nm. We marked this faster‐rising signal as the LE. Subsequently, excitons dissociated to CT states and further separated into electron and hole polaron (or charge separation state, CS), while the latter moved to the donor region inducing the ground state blenching of the donor. We observed the later rising of donor GSB (≈ 550–650 nm) which is the signal of hole transfer. We track the hole polaron signal without the effects of exciton GSB and PIA (610–620 nm, 1020–1030 nm) which also lie in the range of donor GSB and PIA. Accordingly, the signals of 610–620 nm region and 1020–1030 nm region are used to describe the polaron dynamics, while those of 830–840 and 920–930 nm parts are presented to compare the change of singleton decay. Observably, the hole transfer speed and polaron generation kinetics of the two blend systems are almost identical, consistent with their similar JSC, and slightly slower polaron decay of PM6:BTP‐eC9‐4ClO may indicate its suppressed recombination, probably supporting the boosted FF from 79.1% to 80.3%. Certainly, it should be noted that the comprehensive consideration of both TAS and TPV reveals the recombination kinetics in all time scales which closely corrected to the device performance, where PM6:BTP‐eC9‐4ClO possesses longer lifetime in TAS and TPV. Furthermore, the exciton diffusion length of two SMAs is evaluated by fitting TAS data under different pump fluences based on the exciton‐exciton annihilation (EEA) model.^[^ ^67^, ^68^ ^]^ The analyzed results given in **Table** **3** implies BTP‐eC9‐4ClO's exciton has a higher probability to reach the donor/acceptor interface for the next step: splitting. This advantage is beneficial to promote charge generation efficiency. Here no significant difference is observed, which might be due to BTP‐eC9‐based film already containing the ideal exciton dissociation process.

**Table 3:** Exciton diffusion parameters.

Materials	κ [10^−3^ ps^−1^]	α [nm^3^ ps^−1^]	τ	D [nm^2^ ps^−1^]	*L*_D_ [nm]
BTP‐eC9	10.42	293.7	96.3	7.22	26.36
BTP‐eC9‐4ClO	9.8	359.6	101.8	8.95	30.18

**Figure 6:**
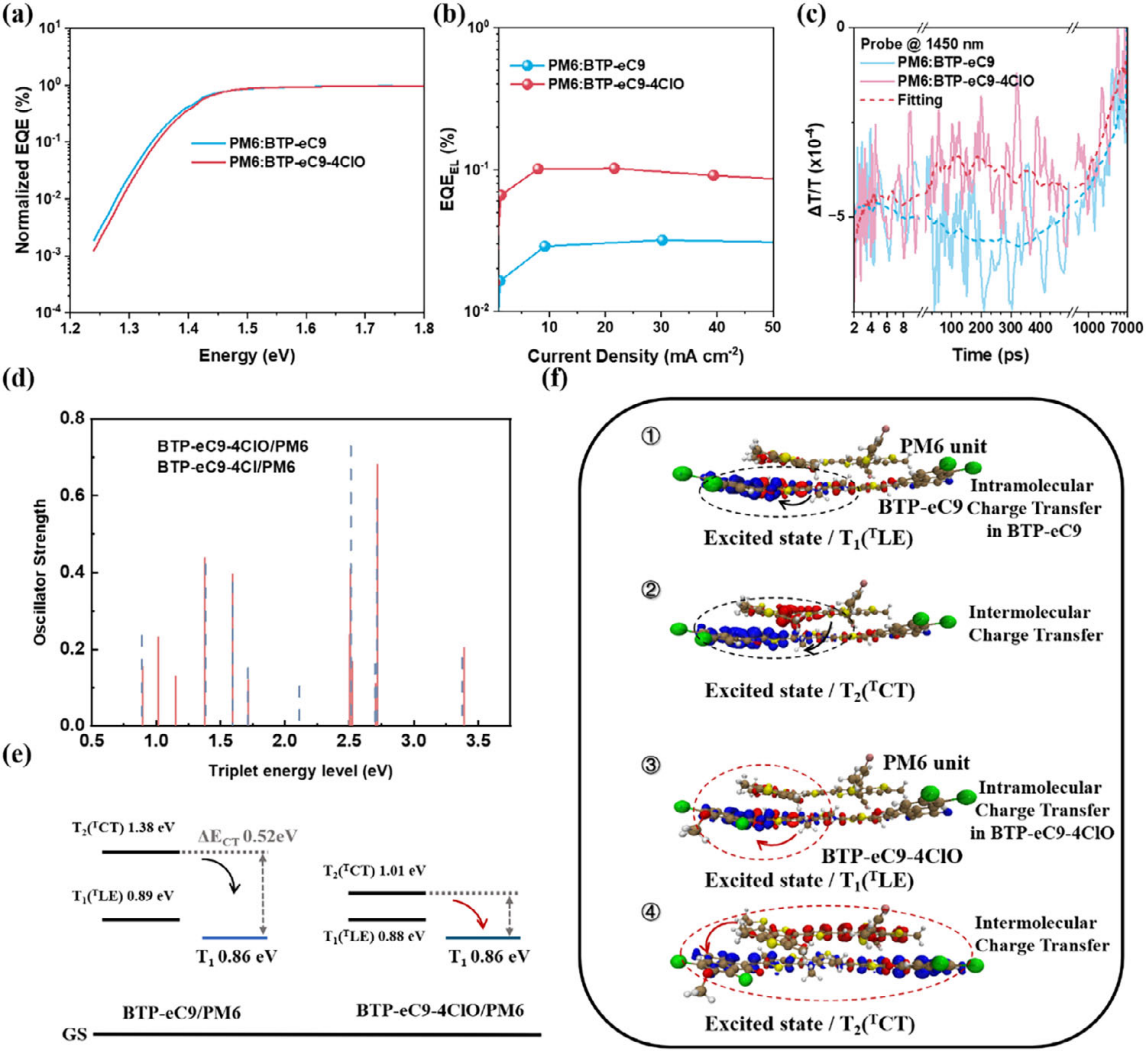
a) FTPS‐EQE spectra. b) EQE_EL_ results. c) triplet formation kinetics. Schematic diagram on the energy alignment of triplet excited states d,e). f) Calculated electron (blue) and hole (red) distribution of the T_1_ and T_2_ in the D:A dimers with eC9‐4Cl (①,②) and eC9‐4ClO (③,④).

The most significantly enhanced parameter is *V*_OC_ based on an almost identical bandgap, indicative of a successfully suppressed energy loss. The Fourier transform photocurrent spectroscopy (FTPS), EQE, and EQE‐electroluminescence experiments on devices are carried out.^[^ ^69^, ^70^ ^]^ According to **Figure** **6**a,b, the energy loss is reduced to 0.530 eV for PM6:BTP‐eC9‐4ClO from 0.555 eV for PM6:BTP‐eC9. This is mainly attributed to the minimized non‐radiative loss from 0.202 to 0.179 eV (Table S6, Supporting Information). Therefore, revealing why the significantly suppressed non‐radiative loss in PM6:BTP‐eC9‐4ClO is the key point to understanding the device performance enhancement.

First, the triplet state formation process is paid attention to by TAS analysis, as the triplet state contributes a lot to non‐radiative decay. As shown by Figure 6c, the tripletons in PM6:BTP‐eC9‐4ClO blend film are indeed clearly at a lower population. More insights on triplet formation suppression and non‐radiative decay channel reduction are gained by theoretical methods, whose analyzing results are displayed in Figure 6d–f. As shown in Figure 6d, the energy level of dimer packing in the triplet excited state of BTP‐eC9/PM6 and BTP‐eC9‐4ClO/PM6. The geometric structures of the triplet excited states are optimized by time‐dependent density functional theory (TD‐DFT). As a result of quantum chemical computations, BTP‐eC9/PM6 and BTP‐eC9‐4ClO/PM6 have similar first triplet localized excited state energy T_1_ (^T^LE) (Figures 6c and 1 and 3), which are 0.89 and 0.88 eV respectively. The lowest‐lying triplet levels of acceptors are considered as the T_1_ states.^[^ ^71^, ^72^ ^]^ It should be noted that these T_1_ (^T^LE) dimers have a close value to T_1_ (0.86 eV), corresponding to the localized excited state (LE) of the dimer originating from the acceptor monomer, indicated by the blue line in Figure 6e.

The triplet excited charge transfer state T_2_ (^T^CT) is defined as the excited state where holes transfer electrons from donor to acceptor (Figures 6c and 2 and 4). Compared with eC9‐4Cl/PM6, the intermolecular charge transfer of eC9‐4ClO/PM6 is that holes are generated in the entire donor molecule PM6, and the electrons are more delocalized in the acceptor molecule eC9‐4ClO. The energy of the T_2_ (^T^CT) of eC9‐4ClO/PM6 (1.01 eV) is significantly reduced compared with eC9‐4Cl/PM6 (1.38 eV). This trend predicted by the quantum chemical calculation is instructive for understanding the electron delocalization caused by the introduction of oxygen‐based alkyl chains within the acceptor end group. The relatively large electronegativity of oxygen may modify the packing structure at the D/A interface and lower the energy of triplet states by mixing ^T^LE and ^T^CT.^[^ ^73^, ^74^ ^]^

To gain more insight, we define the energy difference between the delocalized electronic state of the dimer and the localized electronic state of the monomer as the charge transfer energy ΔE_CT_. EC9‐4ClO/PM6 has a relatively low CT energy barrier, the dynamic equilibrium between the populations at the ^T^CT to T_1_ states helps the charge transfer from donor to acceptor, and more importantly, suppresses the energy loss of the triplet excited state in the eC9‐4ClO/PM6 system.^[^ ^75^ ^]^

## 3 Conclusion

In summary, a new SMA called BTP‐eC9‐4ClO is reported in this work, inspired by our previous success in constructing a methoxylated terminal group. Consequently, the binary OSC based on PM6:BTP‐eC9‐4ClO yields over 20% PCE with nonhalogenated solvent processing, which is also certified by an independent institution. The efficiency increase is mainly attributed to minimized non‐radiative loss, without sacrificing absorption or charge transport. Further investigations reveal that the suppressed triplet formation, driven by oxygen vibration‐induced ^T^LE and ^T^CT mixing. This work addresses several issues for the OSC field: i) the unsatisfying nonhalogenated solvent processed device efficiency; ii) the asymmetric end group contained SMA's sacrificed absorption range; iii) lacking underlying mechanism understanding of non‐radiative loss reduction engineering from the perspective of molecular structure.

## Conflict of Interest

The authors declare no conflict of interest.

## Author Contributions

R.M., B.Z., Y.H., and Y.L. contributed equally to this work. R.M. contributed to project administration, conceptualization, investigation, formal analysis, validation, original draft writing, and methodology. B.Z. focused on conceptualization, investigation, formal analysis, and original draft writing. Y.H. was involved in methodology, formal analysis, and original draft writing. Y.L. contributed to investigation, formal analysis, methodology, and original draft writing. Z.L. provided resources, supervision, methodology, and reviewed and edited the writing. J.W. and H.Y. provided resources; and G.L. contributed resources, supervision, and funding acquisition.

## Supporting information



Supporting Information

## Data Availability

The data that support the findings of this study are available from the corresponding author upon reasonable request.
