# Supplementary material for: Triplet State Suppression for Energy Loss Reduction in 20% Nonhalogenated Solvent Processed Binary Organic Solar Cells
Source: Adv Mater. 2025 Mar 16;37(17):2500861. doi: 10.1002/adma.202500861 (PMC12038535; doi:10.1002/adma.202500861)
Supplement: Supplementary file 1 — Supporting Information [file ADMA-37-2500861-s001.docx]

**Supplementary Information:**

**Triplet State Suppression for Energy Loss Reduction in 20% Non-halogenated Solvent Processed Binary Organic Solar Cells**

Ruijie Ma*^1,5^, Bosen Zou^2,5^, Yongmin Luo^3,5^, Yulong Hai^3,5^, Zhenghui Luo^4^* Jiaying Wu^3^, He Yan^2^, Gang Li^1^*

^1^Department of Electrical and Electronic Engineering, Research Institute for Smart Energy (RISE), Photonic Research Institute (PRI), The Hong Kong Polytechnic University, Hong Kong, 999077, China

Email: ruijie.ma@polyu.edu.hk; gang.w.li@polyu.edu.hk

^2^Department of Chemistry Department of Chemistry and Hong Kong Branch of Chinese National Engineering Research Center for Tissue Restoration and Reconstruction, The Hong Kong University of Science and Technology, Clear Water Bay, Hong Kong, 999077, China

^3^The Hong Kong University of Science and Technology, Function Hub, Advanced Materials Thrust, Nansha Guangzhou, 511400, China

^4^Guangdong Provincial Key Laboratory of New Energy Materials Service Safety, Shenzhen Key Laboratory of New Information Display and Storage Materials, College of Materials Science and Engineering, Shenzhen University, Shenzhen, 518060, China

Email: zhhuiluo@szu.edu.cn

^5^Equal contributor

**Synthesis**

**Figure S1.** Synthesis of **IC-2ClOMe** and **BTP-eC9-4ClO**.

Synthesis of **Compound 2**

**Compound 1** (3 g, 13.6 mmol) was dissolved in distilled tetrahydrofuran (20 mL), and then 2.0 M lithium diisopropylamide in hexane (27 mL, 54 mmol) was added slowly under nitrogen. The reaction was stirred at -78℃ for 3 h and then carbon dioxide gas was added. The reaction mixture was returned to room temperature slowly and stirred overnight. The mixture was poured into water, and acidified to pH 1–2 by addition of the diluted hydrochloric acid and extracted with ethyl acetate for three times. The combined organic phase was washed with water. Then the solution was dried over sodium sulfate and concentrated under reduced pressure. The residue as light yellow solid was used directly without further purification.

Synthesis of **Compound 3**

**Compound 2** (2.5 g, 9.4 mmol) was dissolved in acetic anhydride (15 mL), the reaction was stirred at 140 °C refluxed for 4 h. Then reaction mixture was cooled to room temperature, triethylamine (7.5 mL) and tert-butyl acetoacetate (2.96 g, 18.8 mmol) were added dropwise and the reaction was stirred at 75 °C overnight. The reaction mixture was poured over ice with diluted hydrochloric acid and extracted with dichloromethane, The combined organic phase was washed with water for three times. Then the solution was dried over sodium sulfate and concentrated under reduced pressure. The **Compound 3** as light brown solid was used directly without further purification.

Synthesis of **IC-2ClOMe**

**Compound 3** (1.5 g, 6.1 mmol), malononitrile (1.62 g, 12.2 mmol) were dissolved in 20 mL absolute ethanol, and then anhydrous sodium acetate (1.01 g, 12.2 mmol) was added while stirring under room temperature. After 8 h, the mixture was poured into water, and acidified to pH 1–2 by addition of the hydrochloric acid. Then reaction mixture was extracted by dichloromethane three times and dried over sodium sulfate. The crude product was purified by silicon chromatography with dichloromethane to get pure product **IC-2ClOMe** (697 mg, 39%). **^1^H NMR** (400 MHz, CDCl_3_) *δ* 8.60 (s, 1H), 4.05 (s, 3H), 3.76 (s, 2H). **^13^C NMR** (400 MHz, CDCl_3_) *δ* 190.78, 162.74, 158.86, 139.22, 137.96, 135.98, 128.22, 125.75, 111.76, 111.67, 79.73, 61.67, 44.09. MS (ESI) m/z calcd. for (C_13_H_6_Cl_2_N_2_O_2_): 293.10. Found: 338.95.[M+2Na^+^].

Synthesis of **BTP-eC9-4ClO**

**BTP-eC9-CHO** (200 mg, 0.185 mmol), **IC-2ClOMe** (54.2 mg, 0.185 mmol), **IC-2Cl** (48.7 mg, 0.185 mmol) were dissolved in absolute chloroform (10 mL), and pyridine (1.5 mL) were added. The mixture was deoxygenated with nitrogen for 30 min and then refluxed for 4 h. After cooling to room temperature, the mixture was poured into methanol (100 mL) and filtered. The residue was purified by column chromatography on silica gel using petroleum ether/dichloromethane (1:1.5) as eluent, yielding a dark blue solid and recrystallization through Methanol / Dichloromethane for two times to obtain **BTP-eC9-4ClO** (103.9 mg, 35%). **^1^H NMR** (400 MHz, CDCl_3_) δ 9.16 (s, 1H), 9.15 (s, 1H), 8.77 (s, 1H), 8.70 (s, 1H), 7.95 (s, 1H), 4.99 – 4.62 (m, 4H), 4.06 (s, 3H), 3.22 (t, *J* = 6.5 Hz, 4H), 2.23 – 2.02 (m, 2H), 1.94 – 1.80 (m, 4H), 1.53 – 0.52 (m, 74H). **^13^C NMR** (400 MHz, CDCl_3_) δ 186.17, 185.13, 158.72, 157.62, 157.50, 154.09, 153.98, 147.50, 145.31, 145.29, 139.51, 139.15, 138.75, 137.91, 137.60, 137.20, 136.41, 136.36, 136.08, 135.89, 135.78, 135.51, 134.37, 134.21, 133.62, 133.49, 132.12, 131.21, 130.99, 127.61, 126.90, 125.30, 124.95, 120.44, 119.89, 115.21, 115.07, 114.75, 114.59, 113.71, 113.50, 68.69, 67.94, 61.47, 39.23, 31.87, 31.63, 31.54, 31.23, 30.49, 30.35, 29.84, 29.46, 29.31, 22.86, 22.83, 22.80, 22.67, 22.48, 14.12, 14.06, 14.03, 13.98, 13.78, 13.73. MS (ESI) m/z calcd. for (C_87_H_96_Cl_4_N_8_O_3_S_5_): 1603.88. Found: 1603.50.

**Figure S2.** ^1^H NMR spectrum of **IC-2ClOMe** (400 MHz, CDCl_3_).

**Figure S3**. ^13^C NMR spectrum of **IC-2ClOMe** (400 MHz, CDCl_3_).

**Figure S4**. MS spectrum of **IC-2ClOMe (**C_13_H_6_Cl_2_N_2_O_2_Na_2_**)**.

**Figure S5.** ^1^H NMR spectrum of **BTP-eC9-4ClO** (400 MHz, CDCl_3_).

**Figure S6**. ^13^C NMR spectrum of **BTP-eC9-4ClO** (400 MHz, CDCl_3_).

**Figure S7**. MS spectrum of **BTP-eC9-4ClO**.

**Figure S8**. TGA measurement of **BTP-eC9-4ClO**.

**Table S1**. Calculated parameters for IP (100) peak of neat films.

| Materials | Peak (Å^-1^) | d-spacing (Å) | FWHM (Å^-1^) | CL (Å) |
| --- | --- | --- | --- | --- |
| PM6 | 0.29 | 22.1 | 0.109 | 51.9 |
| BTP-eC9 | 0.39 | 16.2 | 0.100 | 56.6 |
| BTP-eC9-4ClO | 0.39 | 16.0 | 0.102 | 55.4 |

**Table S2**. Calculated parameters for OOP (010) peak of neat films.

| Materials | Peak (Å^-1^) | d-spacing (Å) | FWHM (Å^-1^) | CL (Å) |
| --- | --- | --- | --- | --- |
| PM6 | 1.61 | 3.91 | 0.416 | 13.6 |
| BTP-eC9 | 1.70 | 3.69 | 0.359 | 15.8 |
| BTP-eC9-4ClO | 1.71 | 3.68 | 0.357 | 15.8 |


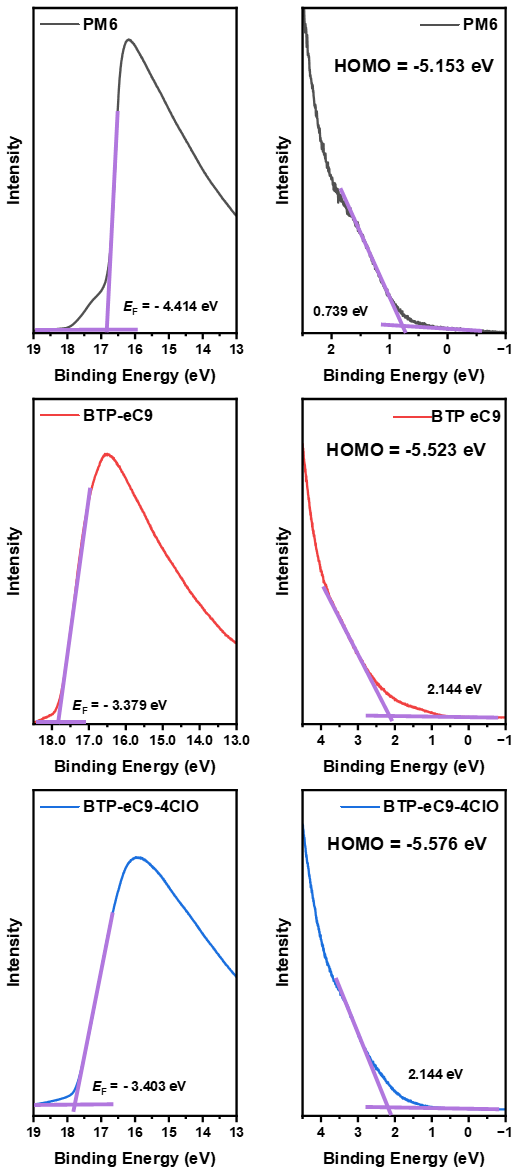


**Figure S9**. The UPS measurement results.

**Table S3**. Calculated parameters for IP (100) peak of blend films.

| Active layers | Peak (Å^-1^) | d-spacing (Å) | FWHM (Å^-1^) | CL (Å) |
| --- | --- | --- | --- | --- |
| PM6:BTP-eC9 | 0.30 | 21.3 | 0.072 | 78.5 |
| PM6:BTP-eC9-4ClO | 0.29 | 21.2 | 0.067 | 84.4 |

**Table S4**. Calculated parameters for OOP (010) peak of blend films.

| Active layers | Peak (Å^-1^) | d-spacing (Å) | FWHM (Å^-1^) | CL (Å) |
| --- | --- | --- | --- | --- |
| PM6:BTP-eC9 | 1.69 | 3.72 | 0.346 | 16.3 |
| PM6:BTP-eC9-4ClO | 1.70 | 3.71 | 0.345 | 16.4 |


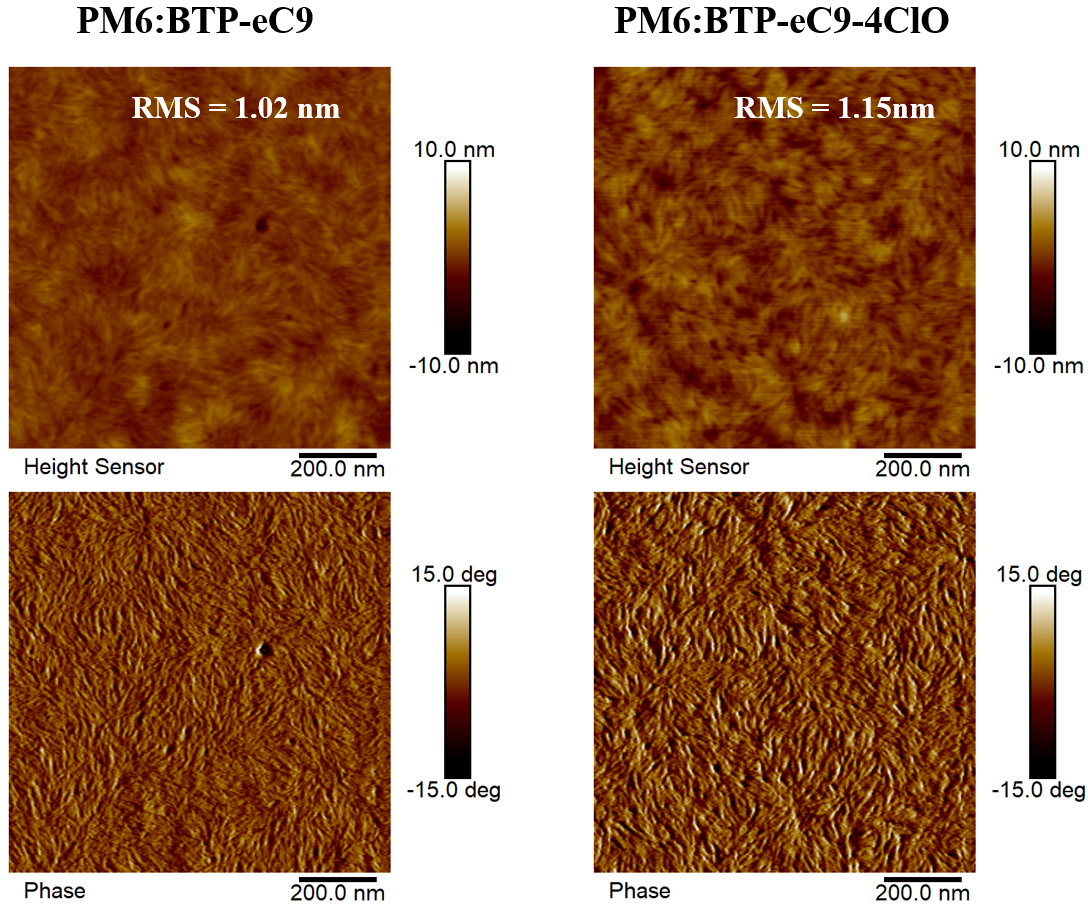


**Figure S10**. AFM height and phase images of PM6:BTP-eC9 and PM6:BTP-eC9-4ClO.


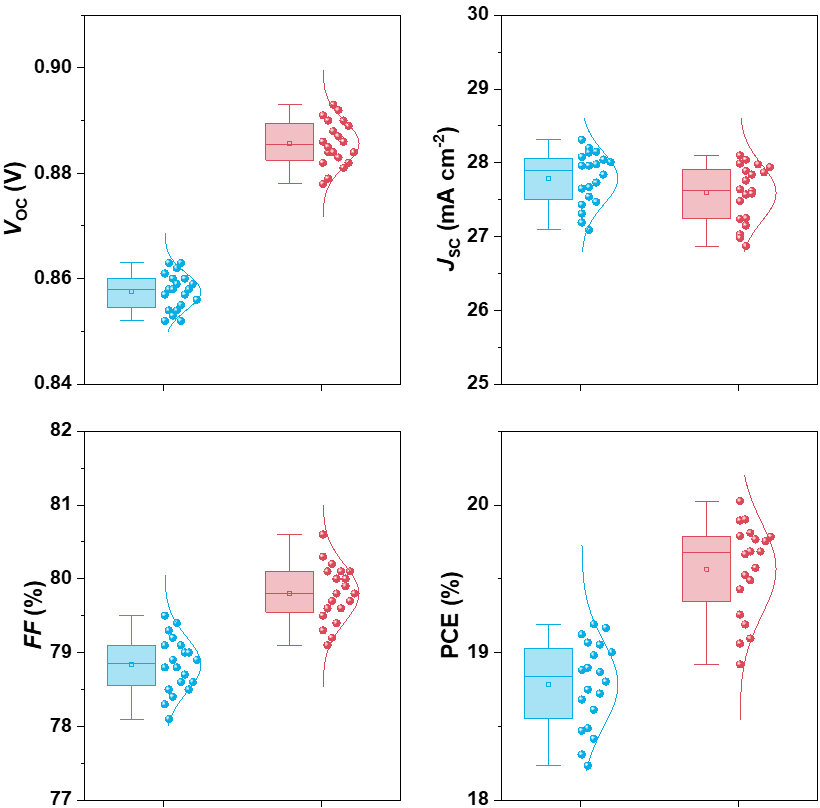


**Figure S11.** Statistic plot of photovoltaic parameters.


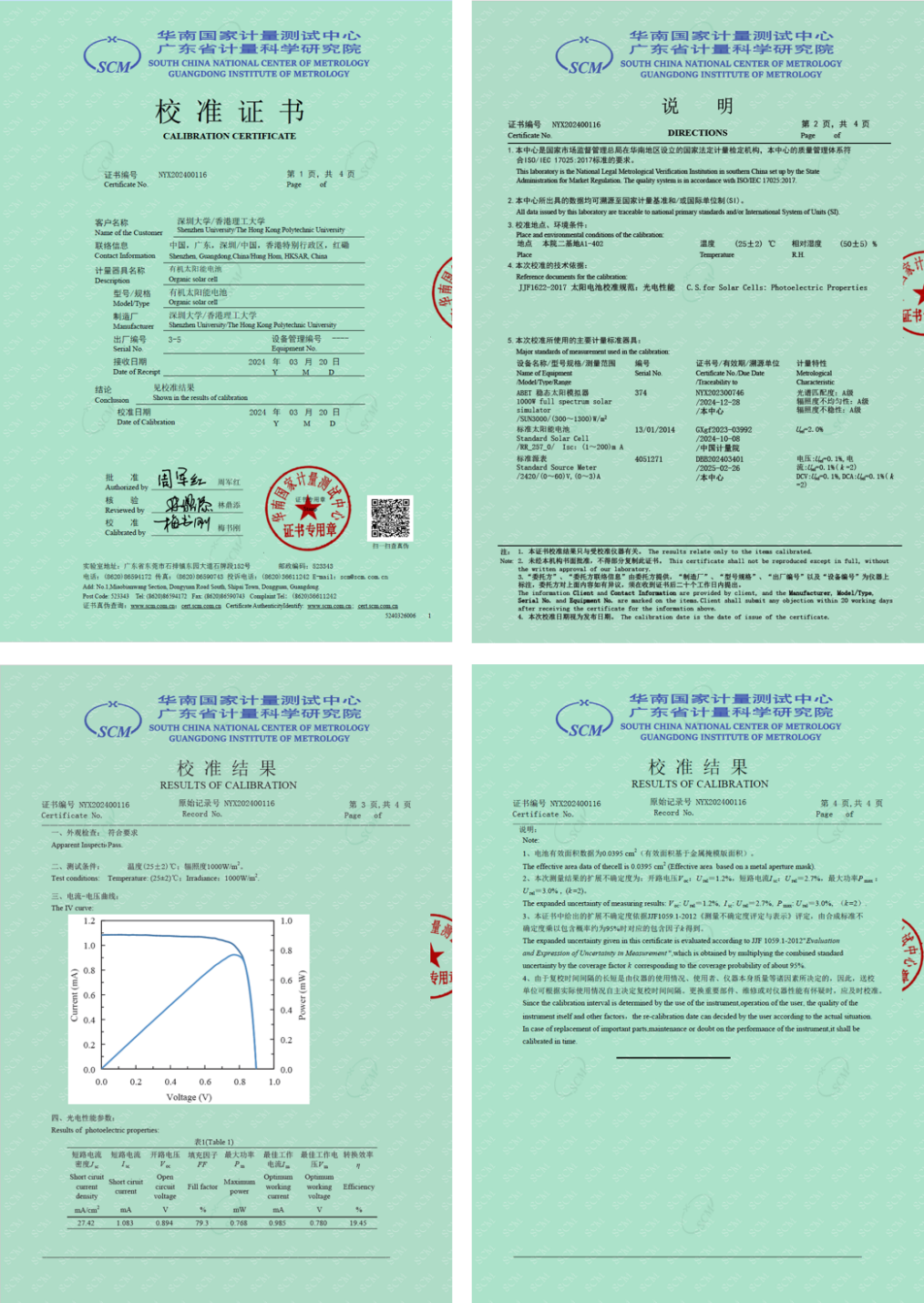


**Figure S12.** Certification report.

**Table S5.** Efficiency Summary of Non-halogenated Solvent Cast OSCs

| **Reference (DOI)** | **Blend** | **V_OC_ (V)** | ***J*_SC_ (mA cm^-2^)** | ***FF* (%)** | **PCE (%)** |
| --- | --- | --- | --- | --- | --- |
| 10.1016/j.joule.2020.07.028 | Binary | 0.856 | 24.94 | 75.5 | 16.1 |
| 10.1002/adfm.202107827 | Ternary | 0.851 | 26.75 | 80 | 18.25 |
| 10.1038/s41560-021-00923-5 | Quaternary | 0.85 | 27.12 | 75.75 | 17.41 |
| 10.1039/D2EE00639A | Binary | 0.84 | 26.77 | 76.33 | 17.15 |
| 10.1002/aenm.202101768 | Ternary | 0.82 | 28.15 | 77.8 | 18 |
| 10.1002/adfm.202107567 | Binary | 0.82 | 26.2 | 74.3 | 16 |
| 10.1016/j.joule.2021.02.010 | Quaternary | 0.86 | 26.33 | 77 | 17.43 |
| 10.1039/D0EE02034F | Binary | 0.854 | 26.1 | 77.7 | 17.33 |
| 10.1039/D1TA07046K | Binary | 0.85 | 25.94 | 73 | 16.11 |
| 10.1002/adma.202107659 | Ternary | 0.843 | 26.83 | 77.43 | 17.54 |
| 10.1002/aenm.202302273 | Binary | 0.858 | 27.5 | 80.5 | 19 |
| 10.1016/j.joule.2023.09.001 | Binary | 0.896 | 25.75 | 79.3 | 19.01 |
| 10.1016/j.cej.2022.139496 | Ternary | 0.842 | 27.14 | 78.28 | 17.89 |
| 10.1016/j.matchemphys.2022.125971 | Binary | 0.83 | 26 | 73.3 | 16.1 |
| 10.1016/j.nanoen.2022.107574 | Binary | 0.87 | 25.6 | 77.9 | 17.4 |
| 10.1016/j.nanoen.2021.106678 | Binary | 0.84 | 25.6 | 76.8 | 16.5 |
| 10.1002/solr.202300029 | Binary | 0.881 | 25.82 | 76.88 | 17.49 |
| 10.3390/molecules27175739 | Binary | 0.906 | 24.3 | 76.5 | 16.8 |
| 10.1002/eom2.12436 | Binary | 0.87 | 27 | 79 | 18.6 |
| 10.1002/adma.202105301 | Binary | 0.806 | 26.29 | 76.2 | 16.16 |
| 10.1039/D2TC03838B | Binary | 0.811 | 26.14 | 77.3 | 16.37 |
| 10.1016/j.xcrp.2021.100517 | Binary | 0.85 | 25.32 | 75 | 16.04 |
| 10.1002/aenm.202203452 | Binary | 0.851 | 26.68 | 76.52 | 17.38 |
| 10.1016/j.cej.2023.142178 | Binary | 0.909 | 24.54 | 78.9 | 17.6 |
| 10.1002/advs.202302376 | Binary | 0.84 | 28 | 77.2 | 18.2 |
| 10.1002/adfm.202214361 | Binary | 0.83 | 26.1 | 76.99 | 16.81 |
| 10.1002/anie.202303551 | Binary | 0.897 | 26.6 | 76.56 | 18.27 |
| 10.1002/aenm.202203465 | Binary | 0.84 | 25.24 | 76.24 | 16.17 |
| 10.1038/s41467-023-41978-0 | Binary | 0.893 | 26.78 | 79.6 | 19.04 |
| 10.1002/aenm.202300904 | Ternary | 0.94 | 25.5 | 75.3 | 18.1 |
| 10.1002/adma.202208926 | Ternary | 0.931 | 24.5 | 79.6 | 18.2 |
| 10.1002/adma.202308334 | Binary | 0.93 | 25.95 | 77.26 | 18.72 |
| 10.1039/D2EE01727J | Binary | 0.886 | 25.12 | 76.64 | 17.06 |
| 10.1007/s11426-023-1608-6 | Binary | 0.885 | 26.25 | 75.3 | 17.5 |
| 10.1002/adma.202302946 | Binary | 0.87 | 26.4 | 80.41 | 18.4 |
| 10.1039/D3EE00294B | Ternary | 0.855 | 27.86 | 80.2 | 19.1 |
| 10.1007/s40820-023-01241-z | Ternary | 0.857 | 28.31 | 79.8 | 19.24 |
| 10.1002/anie.202404297 | Ternary | 0.89 | 27.56 | 80.8 | 19.82 |
| **This work** | Binary | 0.891 | 27.99 | 80.3 | 20.03 |


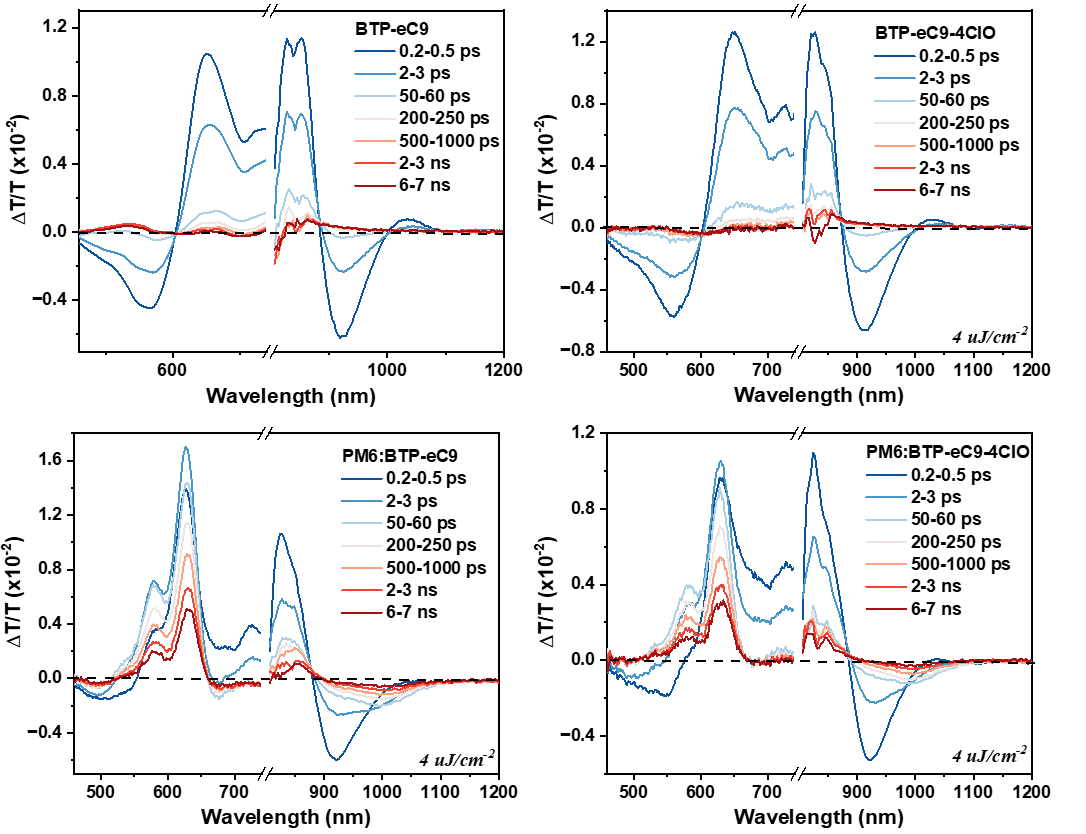


**Figure S13.** Time scale spectral lines of TAS results.

**Characterization**

UV-vis absorption spectra were measured using a Shimadzu UV-2500 recording spectrophotometer. AFM measurements were obtained by using a Dimension Icon AFM (Bruker) in a tapping mode. TEM was recorded on a JEOL JEM-2100 operated at 120kV. UPS measurements were done using ULVAC-PHI multitechnique surface analysis system (PHI VersaProbe4) with He(I) (21.22eV) energy source and using a step resolution of 5 meV. A 10 V bias was applied during the measurements. 2D GISAXS/GIWAXS measurement was performed on an XEUSS 3.0 UHR SAXS/WAXS system (XENOCS, France). A Eiger2 R 1M 2-dimensional detector with 0.075 mm×0.075 mm active pixels were utilized in integration mode. The sample-to-detector distance is settled at 100/2000 mm for GIWAXS/GISAXS measurement. The precise sample-to-detector distance was determined with a silver behenate standard. The Cu incident X-ray (8 KeV) with a 0.9 mm×0.9 mm/0.5 mm×0.5 mm spot provided large enough q space. 1D GIWAXS patterns was corrected to represent real qr and qz axis with the consideration of missing wedge. The critical incident angle was determined by the maximized scattering intensity from sample scattering with negligible contribution from underneath layer scattering. The incident angle scattering was collected at 0.2°, which renders the incident X-ray as an evanescent wave along the top surface of thin films. The samples for GIWAXS/GISAXS test were prepared by casting solution onto silicon wafer substrates (ca. 15 mm×15 mm), and the active layers were prepared using exactly the same concentration and same procedures as those for J–V measurements.

To quantify the micro-nano structures of photovoltaic blend films, the 1D GISAXS profiles (Yonada peak, see Figure 3a) were fitted via a universal model expressed in equation 1.

$$I\left( q \right)=\frac{A_{1}}{{[1+({q\xi)}^{2}]}^{2}}+A_{2}\left( P\left( q,R \right) \right)S\left( q,R,\eta,D \right)+B (1)$$

The first term of the equation is the so-called Debye-Anderson-Brumberger (DAB) term, where q is the scattering wave vector, A_1_ is an independent fitting parameter, and ξ is the average correlation length of the polymer-rich domain. The second term of the equation is assigned to the Fractal model, which means the occupation of fractal-like structure of the acceptor-rich domain. Notably, DAB term describes the domain with more PM6 while fractal-liked term describes the large amount of acceptor. P(q) is related to the form factor of primary acceptor particles (approximated by spherical shape of radius $R$ here, and S(q) is the fractal structure factor, describing the interaction between primary acceptors in this fractal-like aggregation system. The constant B is due to incoherent scattering background. S(q) is given in equation 2. The $\eta$ is the correlation length of the fractal-like network (or domain) formed by the aggregation of primary crystalline particles. D is the fractal dimension. $R$ is the mean radius of primary crystalline particles. The domain size of this network or domain is approximately characterized by $R_{g}$, where $R_{g}$ is the Guinier radius of this fractal-like network (see equation 3).[1]

$$S\left( q,R,\eta,D \right)=1+\frac{\sin[\left( D-1 \right){tan}^{-1}(q\eta)}{\left( qR \right)D}\frac{D\Gamma(D-1)}{[1+\frac{1}{\left( q\eta\right)^{2}}]\frac{D-1}{2}} (2)$$

$$R_{g}=\sqrt{\frac{D(D+1)}{2}} \eta(3)$$

**Solar cell fabrication and characterization**

Solar cells were fabricated in a conventional device configuration of ITO/ Me-4PACz/ PEDOT:PSS/ active layers/ PFN-Br/Ag. The ITO substrates (~94.5% transmittance) were first scrubbed by detergent and then sonicated with deionized water, acetone and isopropanol subsequently, and dried overnight in an oven. The glass substrates were treated by UV-Ozone for 10 min before use. The Me-4PACz deposition is identical to the literature. PEDOT:PSS (standard Hareus Al 4083 solution) was spin-cast onto the ITO substrates at 6500 rpm for 30 s, and then dried at 160 °C for 15 min in air. The blend of PM6:acceptor (BTP-eC9 or BTP-eC9-4ClO) (1:1.2 in weight) blends were dissolved in o-XY (10 mg mL^-1^ donor concentration), with BDCB (10mg/ml) as additive, and stirred on a 100 °C hotplate for 30 mins a nitrogen-filled glove box. The blend solution was spin-cast at 2000 rpm for 50 s onto PEDOT:PSS films followed by a temperature anealing of 100°C for 1 min. PFN-Br thin layers were coated on the active layer with 3000 rpm (0.5 mg mL^-1^), followed by the deposition of Ag (100 nm) (evaporated under 1×10^-3^ Pa through a shadow mask). The optimal active layer thickness measured by a Bruker Dektak XT stylus profilometer was about 105 nm. The current density-voltage (J-V) curves of devices were measured using a Keysight B2901A Source Meter in glove box under AM 1.5G (100 mW cm^-2^) using a Enlitech solar simulator. The device contact area was 0.042 cm^2^, device illuminated area during testing was 0.041 cm2, which was determined by a mask. The EQE spectra were measured using a Solar Cell Spectral Response Measurement System QE-R3011 (Enlitech Co., Ltd.). The light intensity at each wavelength was calibrated using a standard monocrystalline Si photovoltaic cell.

**Sensitive-EQE and EL**

The electroluminescence (EL) spectra were acquired using a Kymera-328I spectrograph and an EMCCD purchased from Andor Technology (DU970P). Injection current used for EL was 1 mA cm^-2^. EQE-EL measurements were done using a home-built setup using a Keithley 2400 to inject current to the solar cells. Emission photon-flux from the solar cells was recorded using a Si detector (Hamamatsu s1337-1010BQ) and a Keithley 6482 picoammeter. Sensitive EQE measurements were done using a halogen lamp light source, chopped at a frequency of 173 Hz, a monochromator (Newport CS260), a Stanford SR830 lock-in amplifier, a Stanford SR570 current amplifier, and a set of long pass filters. Lamp intensity was calibrated using a Si detector (Hamamatsu s1337-1010BQ).

**Table S6.** Energy loss parameters.

| **PM6:acceptor** | ***E*_g_ (eV)** | ***EQE*_EL_*@J*_SC_** | ***∆E*_1_ (eV)** | ***∆E*_2_ (eV)** | ***∆E*_3_ (eV)** | ***∆E*_tot_ (eV)** |
| --- | --- | --- | --- | --- | --- | --- |
| BTP-eC9 | 1.416 | 2.88×10^-4^ | 0.255 | 0.098 | 0.202 | 0.555 |
| BTP-eC9-4ClO | 1.421 | 1.01×10^-3^ | 0.255 | 0.096 | 0.179 | 0.530 |

**Transient Absorption Spectroscopy**

Transient absorption measurement was conducted on a commercial pump-probe femtosecond transient absorption (TA) spectrometer Helios (Ultrafast System, USA). Ultrafast laser pulses (800 nm, < 35 fs pulse duration, 7 W) was generated by 1 kHz Ti:Sapphire regenerative amplifier (Astrella, Coherent, USA). 40% of the fundamental pulses (7 W) was used to pump the commercial collinear optical parametric amplifier (TOPAS Prime, Light-Conversion, Lithuania) for generating tunable wavelength pump pulse to 400 nm or 800 nm. The pump beam is chopped at 500 Hz. 15% of the fundamental pulses was routed onto a mechanical delay stage (within 7 ns) and passed through a sapphire crystal to generate supercontinuum probe light (450-750 nm) and a YAG crystal to generate NIR supercontinuum probe light (800 nm-1200 nm). The pump light and probe light were focused on a same spot (2 mm diameter) of the thin films placed on a quartz. Data analysis is performed by Surface Xplorer software. The incident power is measured with a calibrated laser power meter (Newport).

**Calculation Methods**

Gaussian 16 (Revision C.02) code^1^ was used to perform density functional theory (DFT) calculations at the non-empirically tuned B3LYP-D3(BJ)/TZVP^2-4^ level of theory. The side chains of molecular were modelled as methyl groups to reduce the computational cost; while the nature of the side chains is an important factor controlling the molecule packing in the solid state, it has only marginal influence on the intrinsic electronic and optical properties of the π-conjugated backbones. In the calculation of the highest occupied molecular orbital (HOMO) and lowest unoccupied molecular orbital (LUMO) energy levels, the single point energy was calculated under B3LYP-D3(BJ)/Def2TZVP level for high precision calculations. The wavefunction software Multiwfn^5^ and VMD^6^ were used for analyzing the electron and hole distribution.

**Reference**

1. Gaussian 16, Revision C.02, M. J. Frisch, G. W. Trucks, H. B. Schlegel, G. E. Scuseria, M. A. Robb, J. R. Cheeseman, G. Scalmani, V. Barone, G. A. Petersson, H. Nakatsuji, X. Li, M. Caricato, A. V. Marenich, J. Bloino, B. G. Janesko, R. Gomperts, B. Mennucci, H. P. Hratchian, J. V. Ortiz, A. F. Izmaylov, J. L. Sonnenberg, D. Williams-Young, F. Ding, F. Lipparini, F. Egidi, J. Goings, B. Peng, A. Petrone, T. Henderson, D. Ranasinghe, V. G. Zakrzewski, J. Gao, N. Rega, G. Zheng, W. Liang, M. Hada, M. Ehara, K. Toyota, R. Fukuda, J. Hasegawa, M. Ishida, T. Nakajima, Y. Honda, O. Kitao, H. Nakai, T. Vreven, K. Throssell, J. A. Montgomery, Jr., J. E. Peralta, F. Ogliaro, M. J. Bearpark, J. J. Heyd, E. N. Brothers, K. N. Kudin, V. N. Staroverov, T. A. Keith, R. Kobayashi, J. Normand, K. Raghavachari, A. P. Rendell, J. C. Burant, S. S. Iyengar, J. Tomasi, M. Cossi, J. M. Millam, M. Klene, C. Adamo, R. Cammi, J. W. Ochterski, R. L. Martin, K. Morokuma, O. Farkas, J. B. Foresman, and D. J. Fox, Gaussian, Inc., Wallingford CT, 2019. D. J. Fox, Gaussian 16, Revision C.02, Gaussian, Inc., Wallingford CT, (2019).

2. Becke, A. D. A new mixing of Hartree–Fock and local density‐functional theories. J. Chem. Phys. 98, 1372–1377 (1993).

3. Stefan Grimme, Jens Antony, Stephan Ehrlich and Helge Krieg, J. Chem. Phys. 132, 154104 (2010).

4. Benjamin P. Pritchard, Doaa Altarawy, Brett Didier, Tara D. Gibson, Theresa L. Windus. J. Chem. Inf. Model. 59(11), 4814-4820, (2019).

5. Lu, T. & Chen, F. Multiwfn: a multifunctional wavefunction analyzer. J. Comput. Chem. 33, 580–592 (2012).

6.W. Humphrey, A. Dalke, K. Schulten, J. Mol. Graph. 1996, 14, 33.
